# A conserved Lsm8–exosome module maintains RNA splicing fidelity to control fungal stress adaptation and virulence

**DOI:** 10.1007/s44154-026-00285-6

**Published:** 2026-02-10

**Authors:** Yiyi Ren, Haolan Cheng, Xingmin Han, Meiling Guo, Chenghui Xu, Jiayue Yan, Zhiwei Ge, Zhonghua Ma, Yun Chen

**Affiliations:** 1 State Key Laboratory of Rice Biology and Breeding, Zhejiang Key Laboratory of Biology and Ecological Regulation of Crop Pathogens and Insects, Institute of Biotechnology, Zhejiang University, Hangzhou, 310058 China; 2 Analysis Center of Agrobiology and Environmental Sciences, Zhejiang University, Hangzhou, 310058 China

**Keywords:** Lsm2-8 complex, RNA splicing fidelity, RNA exosome, Post-transcriptional regulation, Intron retention, Stress response, Fungal virulence, Mycotoxin biosynthesis, *Fusarium graminearum*

## Abstract

*Fusarium graminearum*, the causal agent of Fusarium head blight (FHB), poses a major threat to global food security by contaminating cereals with the mycotoxin deoxynivalenol (DON). Although transcriptional and protein-level regulation of its stress response and virulence has been extensively studied, the functional significance of mRNA processing in these critical processes remains largely unexplored. Here, we identify Lsm8, a highly conserved core subunit of the nuclear Lsm2-8 complex, as a pivotal regulator linking RNA splicing fidelity to fungal growth, stress adaptation, and virulence. Deletion of *LSM8* disrupted Lsm2-8 assembly and nuclear localization, resulting in widespread intron retention in genes essential for stress signaling (*HOG1*, *ATF1*), development (*GPA1*, *STE12*), and trichothecene biosynthesis. Consequently, osmoadaptation was impaired, sexual reproduction was abolished, and both DON production and virulence were drastically reduced. We further demonstrate that intron-retained transcripts are predominantly degraded by the RNA exosome, revealing a conserved Lsm8–exosome module that maintains splicing fidelity and RNA surveillance. Given the deep evolutionary conservation of Lsm8 across eukaryotes, these findings uncover a fundamental post-transcriptional regulatory layer governing fungal stress response, virulence, and mycotoxin biosynthesis, and highlight RNA-processing factors as universal determinants of virulence and promising antifungal targets across eukaryotic pathogens.

**Graphical Abstract:**

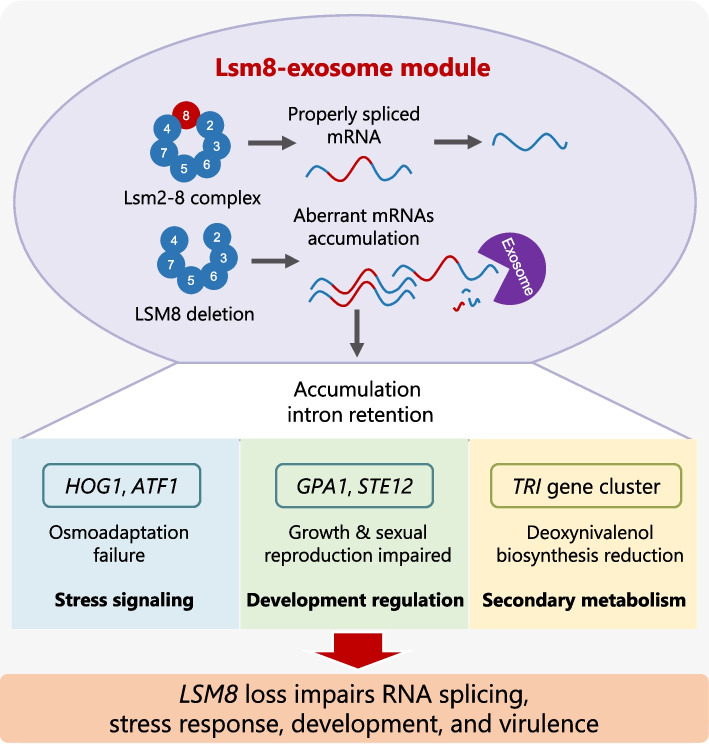

**Supplementary Information:**

The online version contains supplementary material available at 10.1007/s44154-026-00285-6.

## Introduction

In eukaryotes, pre-messenger RNA (pre-mRNA) transcripts are processed by the spliceosome, a highly dynamic macromolecular machinery, to produce mature messenger RNA (mRNA) essential for protein synthesis. Splicing occurs as constitutive splicing, which generates a single transcript from a given precursor, and as alternative splicing (AS), which selects distinct splice sites to expand proteomic and regulatory diversity essential for development and stress adaptation. The spliceosome itself is a large ribonucleoprotein complex with five small nuclear RNAs (snRNAs U1, U2, U4, U5, and U6) and numerous accessory proteins (Matera and Wang 2014; Wilkinson et al. 2020). Within this complex, U1, U2, U4, and U5 snRNAs associate with canonical Sm protein complexes. The Sm proteins are a family of highly conserved RNA-binding proteins that form heptameric rings and are crucial components of the spliceosome, named after their initial discovery as targets of anti-Sm autoantibodies in patients with systemic lupus erythematosus (Tan 1989). In contrast, the catalytic core U6 snRNA specifically binds to the nuclear Lsm (or "Like Sm") heteroheptameric complex, Lsm2-8 (Spiller et al. 2007; Veretnik et al. 2009). The "Like-Sm" designation reflects the structural analogy of these proteins to the classic Sm core, sharing a highly conserved "Sm fold" and the ability to form heptameric rings (Zhou et al. 2014). However, Lsm proteins are functionally distinct from canonical Sm protein. The Lsm protein family forms two distinct, functionally specialized heteroheptameric ribonucleoprotein complexes: the cytoplasmic Lsm1-7 complex and the nuclear Lsm2-8 complex. Both complexes share the Lsm2-7 subunits. However, their distinct compositions and cellular localizations define their specialized functions: the cytoplasmic Lsm1-7 complex, which uniquely contains Lsm1, is primarily involved in mRNA decay, promoting decapping and subsequent 5′ → 3′ exonucleolytic degradation (Montemayor et al. 2020). In contrast, the nuclear Lsm2-8 complex, uniquely possessing Lsm8, is crucial for pre-mRNA splicing (where it primarily binds the 3′-end of U6 snRNA) and nuclear mRNA decay (Tan 1989; Lührmann et al. 1990). The nuclear Lsm2-8 complex specifically recognizes the 3′-oligouridylate sequence (GUUUU) of U6 snRNA (Pannone et al. 2001; Perea-Resa et al. 2012; Montemayor et al. 2020). This protects it from exonucleolytic degradation and facilitates continuous recycling during splicing cycles (Zhou et al. 2014). Beyond its canonical role in U6 snRNA stabilization, the nuclear Lsm2-8 complex has also been implicated in processing other RNA species, including pre-tRNAs, pre-snoRNAs, and pre-rRNAs, as well as pre-mRNA turnover (Kufel et al. 2003, 2004). While the functions of the Lsm2-8 complex in spliceosome dynamics and catalytic cycling are well-documented, its potential regulatory influence on the transcriptional expression of other splicing factors remains largely unexplored. Furthermore, aberrant spliceosome function can lead to pre-mRNA splicing defects, generating abnormal transcripts. These typically activate the nonsense-mediated mRNA decay (NMD) pathway to prevent toxic truncated protein accumulation (Monteuuis et al. 2019; Lash et al. 2024). However, whether and how the Lsm2-8 complex directly interacts with or contributes to the NMD pathway, especially in filamentous fungi, is still unclear.

Genetic studies across diverse eukaryotes confirm a conserved role for Lsm2-8 in fundamental biological processes. In *Saccharomyces cerevisiae*, Lsm8 mutations severely impair the ability of Lsm2-8 complex to bind U6 snRNA, causing pronounced growth defects (Roth et al. 2018). In *Arabidopsis thaliana*, Lsm8 deficiency triggers widespread intron retention (IR) in mRNAs of genes crucial for embryonic development, signal transduction, and protein metabolism. This results in abnormal cotyledon morphology, inhibited root development, early flowering, and impaired seed formation. Moreover, this complex modulates splicing accuracy and efficiency during cold and salt stress, highlighting their role in abiotic stress adaptation (Golisz et al. 2013; Carrasco-López et al. 2017). In *Caenorhabditis elegans*, the Lsm2-8 complex cooperates with exonuclease Xrn2 to degrade transcripts associated with H3K27me3-marked loci. This reinforces Polycomb-dependent transcriptional silencing and ensures normal development (Mattout et al. 2020). Lsm8 deletion leads to severe developmental disorders, including larval arrest, reproductive sterility, and embryonic lethality. *F. graminearum* is a globally significant plant pathogen causing Fusarium head blight (FHB) in cereal crops like wheat and maize. This devastating disease causes substantial yield losses and contaminates infected grains with trichothecene mycotoxins, most notably deoxynivalenol (DON) and zearalenone. This poses severe risks to food and feed safety, human, and animal health (Chen et al. 2019). Previous studies demonstrated the critical importance of other pre-mRNA splicing factors for fungal growth, development, virulence, and DON synthesis in *F. graminearum*. For example, the RNA binding protein FgRbp1 interacts with FgU2AF23 to promote splicing of ribosome-related transcripts, impacting growth and pathogenicity (Wang et al. 2021). Serine/arginine (SR) proteins FgSrp1 and FgSrp2 modulate asexual reproduction and virulence by tuning splicing efficiency (Zhang et al. 2017, 2020). Deletion of the spliceosomal protein kinase FgPrp4 reduces intron splicing efficiency genome-wide and severely impairs development (Gao et al. 2016). Despite these compelling advances, the specific role of the Lsm2-8 complex in *F. graminearum*, and whether it coordinates pre-mRNA splicing with fungal growth, virulence, and mycotoxin production, remains entirely unexplored.

The objectives of this study were to (i) characterize the function of Lsm8, a core subunit of the Lsm2-8 complex, in pre-mRNA splicing efficiency and transcript integrity in *F. graminearum*; (ii) investigate its potential regulatory influence on the transcriptional expression of other splicing factors; (iii) elucidate the precise contributions of Lsm8 to hyphal growth, environmental adaptation, and pathogenicity; (iv) investigate the impact of Lsm8 on the splicing efficiency of trichothecene biosynthetic genes and its subsequent control over DON production; and (v) explore the genetic interactions between Lsm8 and the NMD pathway, as well as RNA degradation pathways, in facilitating aberrant transcript clearance in this significant plant pathogen.

## Results

### Lsm8 is essential for Lsm2-8 complex assembly and nuclear localization in *F. graminearum*

**Fig. 1:**
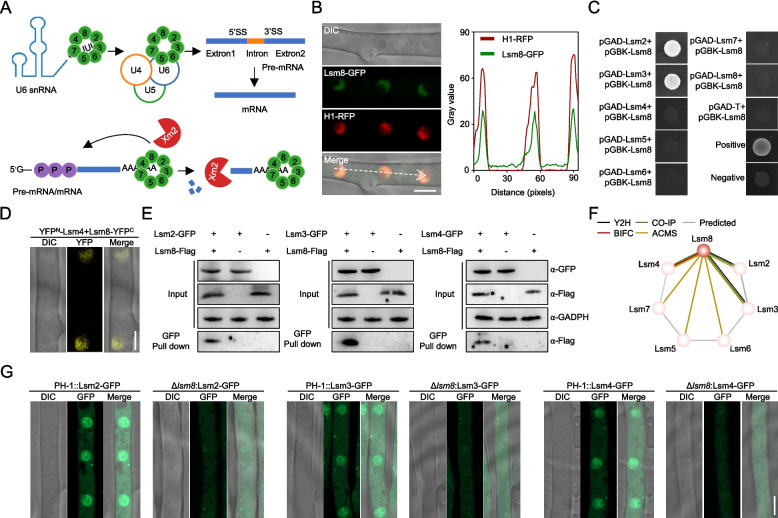
Lsm8 is critical for Lsm2-8 complex assembly and nuclear localization in *F. graminearum*. **A** Schematic representation of the role of Lsm2-8 complex in pre-mRNA splicing and nuclear mRNA decay. **B** Subcellular localization of Lsm8-GFP (green) and nuclear marker Histone H1-RFP (red) in *F. graminearum*. The merged image shows co-localization. A line-scan profile indicates the fluorescence intensity distribution across the arrowed region. Scale bar, 5 µm. **C** Yeast two-hybrid (Y2H) assays demonstrating direct interactions between Lsm8 and Lsm2, Lsm3, and Lsm4. Growth on selective medium (-Leu/-Trp/-His/-Ade) indicates interaction. **D** Bimolecular fluorescence complementation (BiFC) assay showing nuclear interaction between Lsm8-cYFP and Lsm4-nYFP in *F. graminearum* mycelia. YFP fluorescence indicates interaction. Scale bar, 5 µm. **E** Co-immunoprecipitation (Co-IP) experiments validating the in vivo association of Lsm8 with Lsm2, Lsm3, and Lsm4. Western blot analysis confirms the interactions. **F** Proposed model of the Lsm2-8 complex assembly, highlighting Lsm8 as a central subunit. Colored lines indicate experimentally validated interactions, while gray lines denote predicted potential interactions. **G** Subcellular localization of Lsm2-GFP, Lsm3-GFP, and Lsm4-GFP in PH-1 and Δ*lsm8* strains. Confocal microscopy images show diffuse cytoplasmic localization of Lsm-GFP proteins in Δ*lsm8* compared to their nuclear localization in the wild-type. Scale bar, 5 µm

Given the established roles of Lsm protein complexes in RNA metabolism, and in particular the central function of the nuclear Lsm2-8 complex in pre-mRNA splicing (Fig. 1A) (He and Parker 2000), we selected Lsm8 (encoded by FGSG_30019) as a representative subunit to thoroughly investigate the biological functions of this complex in *F. graminearum*. Lsm8's unique specificity to the nuclear Lsm2-8 complex makes it an ideal target for this investigation.

Sequence analysis revealed that Lsm8 contains a canonical Like-Sm domain associated with RNA binding and exhibits high sequence identity to its homologs in *S. cerevisiae* (49.59%) and *Homo sapiens* (65.25%) (Fig. S1A). Phylogenetic analysis further confirmed the deep conservation of Lsm8 across diverse eukaryotes (Fig. S1B). Despite the absence of a predicted canonical nuclear localization signal, an Lsm8-GFP fusion protein clearly co-localized with the nuclear marker Histone H1-RFP, definitively confirming its nuclear localization (Fig. 1B). To elucidate the assembly of the Lsm2-8 complex, we performed a series of protein–protein interaction assays. Yeast two-hybrid (Y2H) assays demonstrated direct interactions between Lsm8 and Lsm2 and Lsm3 (Fig. 1C). Bimolecular fluorescence complementation (BiFC) further revealed a nuclear interaction between Lsm8 and Lsm4 (Fig. 1D). These interactions were validated in vivo by co-immunoprecipitation (Co-IP) experiments, confirming the association of Lsm8 with Lsm2, Lsm3, and Lsm4 (Fig. 1E). Additionally, affinity capture coupled with mass spectrometry (AC-MS), using Lsm8-GFP as bait, successfully co-purified all six other Lsm subunits (Lsm2-7) (Table S2, Fig. S1C). These data collectively indicate that Lsm8 serves as a central architectural subunit facilitating the assembly of the full heteroheptameric Lsm2-8 complex in this fungus (Fig. 1F). Crucially, Lsm8 proved essential for the proper nuclear targeting of other Lsm subunits. Deletion of *LSM8* in strains expressing Lsm2-GFP, Lsm3-GFP, or Lsm4-GFP resulted in the complete loss of their nuclear localization signals, with GFP signals diffusely distributed throughout the cytoplasm (Fig. 1G, Fig. S1D). Taken together, Lsm8 is indispensable for both the assembly and nuclear localization of the Lsm2-8 complex in *F. graminearum*, highlighting its pivotal role in the integrity and function of the complex.

### Lsm8 regulates vegetative growth by impacting RNA metabolism and key biosynthetic pathways

**Fig. 2:**
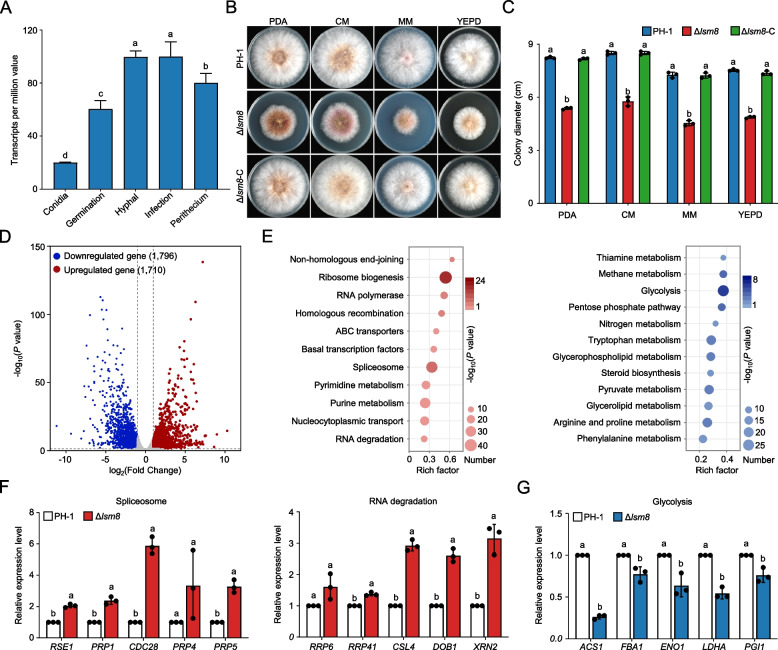
Lsm8 regulates vegetative growth and impacts RNA metabolism and key biosynthetic pathways. **A** Expression of *LSM8* across different life stages, measured as transcripts per million. Samples were collected during conidiation (3 d), conidial germination (12 h), hyphal growth (24 h), plant infection (3 days), and perithecium formation (3 d). **B** Vegetative growth of PH-1, Δ*lsm8*, and Δ*lsm8*-C strains on PDA, MM, CM, and YEPD plates, incubated at 25 °C for 3 days. **C** Quantification of colony diameters for PH-1, Δ*lsm8*, and Δ*lsm8*-C strains. **D** Volcano plot depicting the distribution of differentially expressed genes between PH-1 and Δ*lsm8*. Upregulated genes are marked in red, and downregulated genes are marked in blue. **E** KEGG pathway enrichment analysis of DEGs, highlighting significant changes in Δ*lsm8*. **F** RT-qPCR validation of selected upregulated mRNAs in Δ*lsm8*. **G** RT-qPCR validation of selected downregulated mRNAs in Δ*lsm8*. Data are shown as means ± SD. Different letters in figure indicate a significant difference (*P* < 0.05) based on one-way ANOVA followed by Tukey’s HSD test and unpaired Student’s *t*-test. Data are shown as means ± SD, *n* = 3

Based on our finding that Lsm8 is a core subunit critical for the integrity and nuclear localization of the Lsm2-8 complex, we next investigated its biological roles in *F. graminearum*. Transcriptional expression analysis of the *LSM8* gene, using public available RNA-sequencing (RNA-seq) data, revealed consistently high transcript levels across diverse fungal life cycle stages. These included conidiation, conidial germination, hyphal growth, plant infection, trichothecene biosynthesis induction (TBI), and perithecium formation. Notably, *LSM8* expression was highest during hyphal growth (Fig. 2A), suggesting its critical role in vegetative development and broader involvement in fungal development, pathogenicity, and mycotoxin synthesis. To functionally characterize Lsm8, we generated a deletion mutant Δ*lsm8*. Phenotypic comparisons with the wild-type (WT, PH-1) and complemented (Δ*lsm8*-C) strains revealed that Δ*lsm8* exhibited significantly reduced growth rates on all tested media, including PDA, minimal medium (MM), complete medium (CM), and yeast extract peptone dextrose (YEPD) agar (Fig. 2B, C). These findings confirm Lsm8 as a key regulator of vegetative growth in *F. graminearum*.

To dissect the mechanisms underlying the observed growth defect of Δ*lsm8*, we compared RNA-seq between PH-1 and Δ*lsm8*. RNA-seq analysis revealed substantial transcriptional reprogramming in the mutant, with 1,710 genes significantly upregulated (log_2_FC > 1, FDR < 0.05) and 1,796 genes significantly downregulated (log_2_FC < −1, FDR < 0.05) (Fig. 2D). Kyoto Encyclopedia of Genes and Genomes (KEGG) pathway enrichment analysis of the differentially expressed genes provided critical insights. Upregulated genes were significantly enriched in pathways related to ribosome biogenesis, spliceosome function, RNA degradation, and DNA recombination/repair. This supports the established nuclear roles of Lsm proteins in mRNA splicing and turnover, with the observed upregulation likely representing a compensatory or feedback response to the loss of a key component of this complex. Conversely, downregulated genes were significantly enriched in core metabolic and biosynthetic pathways, such as carbon metabolism (glycolysis, pentose phosphate pathway), nitrogen metabolism (Fig. 2E). This widespread downregulation in metabolic pathways suggests that Lsm8 is crucial for coordinating cellular energy supply and biosynthetic processes, thereby providing a molecular explanation for the severe growth impairment of the mutant. To validate the RNA-seq data, we performed RT-qPCR for selected differentially expressed genes. Ten upregulated genes from RNA splicing and degradation pathways (splicing factors: *RES1, RRP1, CDC28, PRP4, PRP5*; RNA degradation factors: *RRP6, RRP41, CSL4, DOB1, XRN2*) showed consistent and significant upregulation, corroborating the transcriptomic results (Fig. 2F). Similarly, five downregulated genes from the glycolysis pathway (*ACS1, FBA1, ENO1, LDHA, PGI1*) exhibited concordant reductions in expression by RT-qPCR (Fig. 2G). Collectively, these results unequivocally demonstrate that Lsm8 is essential for the vegetative growth of *F. graminearum*. Its absence fundamentally disrupts core RNA metabolic pathways and critical energy-producing and biosynthetic processes, providing a molecular basis for the observed severe growth defects. Notably, the observed transcriptional upregulation of several splicing factors in Δ*lsm8* suggests that the Lsm2-8 complex, beyond its direct involvement in spliceosome assembly, also plays a regulatory role at the transcriptional level, influencing the expression of splicing components.

### Lsm8 controls pre-mRNA splicing fidelity by modulating IR

**Fig. 3:**
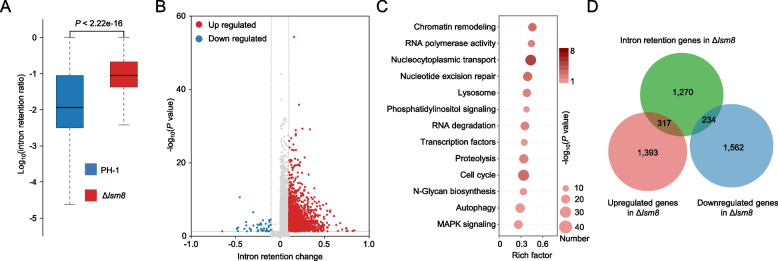
Lsm8 loss leads to widespread intron retention and dysregulation of associated pathways. **A** Box plot comparing intron retention (IR) rates between PH-1 and Δ*lsm8*. **B** Volcano plot illustrating significantly altered intron retention events in Δ*lsm8* compared to PH-1. Introns with significantly increased retention (IR change = |IRΔ*lsm8*-IRWT| ≥ 0.1,* P* < 0.05) are highlighted. **C** KEGG pathway enrichment analysis of the 1,821 genes with significantly retained introns. The analysis indicates enrichment in crucial cellular processes. **D** Overlap analysis of intron-retained genes with differentially expressed genes in Δ*lsm8*

Given that the Lsm2-8 complex interacts with the catalytic core U6 snRNA within the spliceosome (Zhou et al. 2014), we hypothesized that the growth defects of Δ*lsm8* were a direct consequence of impaired pre-mRNA splicing fidelity. To test this hypothesis, we performed a comprehensive comparative analysis of IR events between PH-1 and Δ*lsm8* using strand-specific transcriptome data (reads with counts per million ≥ 1). Our analysis revealed a dramatic and widespread increase in IR in Δ*lsm8*. While a total of 13,982 introns were detected across both strains, the overall IR rate in Δ*lsm8* was profoundly higher than in the WT, with a median IR rate of 0.089 compared to just 0.011 in the WT (*P* < 2.2 × 10^–16^) (Fig. 3A). This indicates that Lsm8 is critical for ensuring the global efficiency and fidelity of splicing in *F. graminearum*. Further detailed analysis identified 2,344 introns from 1,821 genes that exhibited a significant increase in IR compared to the WT (IR change = |IRΔ*lsm8*-IRWT| ≥ 0.1, *P* < 0.05) (Fig. 3B). In contrast, only 45 introns from 40 genes showed a significant decrease in retention, firmly confirming that the loss of Lsm8 primarily impacts splicing negatively. KEGG pathway enrichment analysis of the genes with significantly retained introns showed that they are enriched in pathways crucial for cellular function and integrity, including chromatin remodeling, RNA polymerase activity, nucleocytoplasmic transport, and nucleotide excision repair (Fig. 3C).

We further investigated the relationship between these splicing defects and global gene expression. Among the 1,821 genes with significant IR, we found that 317 genes had significantly upregulated transcription levels and 234 were significantly downregulated. Strikingly, most of these genes (1,270) showed no significant change in their overall mRNA transcript level (Fig. 3D). This is a crucial finding, as it demonstrates that while some transcriptional changes occur (Fig. 2D), the primary impact of Lsm8 loss on these genes is at a post-transcriptional level. The retention of these introns in the coding sequences of their respective mRNAs is expected to disrupt the reading frame, leading to the production of non-functional or truncated proteins, or inducing nonsense-mediated decay, ultimately impacting a wide range of essential biological processes. Collectively, these data strongly suggest that Lsm8 acts as a key regulator of splicing fidelity in *F. graminearum*, and its disruption causes extensive and widespread IR in transcripts of key functional genes, leading to the severe phenotypic defects seen in the mutant.

### Lsm8 regulates osmotic stress adaptation by controlling pre-mRNA splicing of HOG-MAPK components

**Fig. 4:**
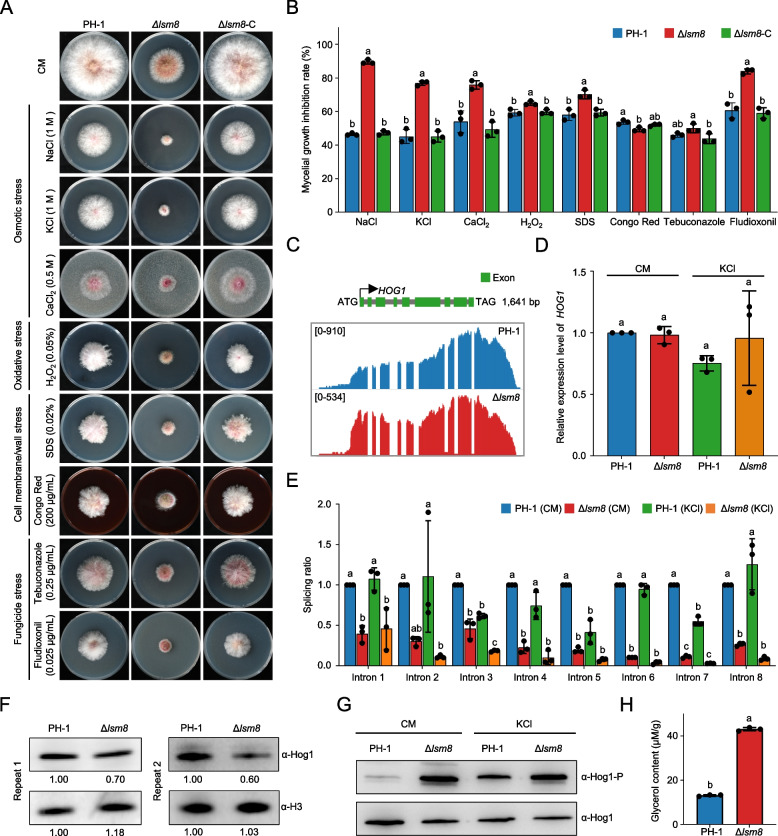
Lsm8 regulates HOG1 splicing and osmotic stress adaptation. **A** Growth phenotypes of PH-1, Δ*lsm8*, and complemented strain Δ*lsm8*-C under various stress conditions. **B** Quantification of colony diameters from (**A**). **C** Integrative Genomics Viewer (IGV) visualization of RNA-seq data showing *HOG1* intron retention in Δ*lsm8* compared to PH-1. **D** RT-qPCR analysis of total *HOG1* mRNA levels in PH-1 and Δ*lsm8* under untreated and osmotic stress (1 M KCl). **E** Splicing efficiency analysis of *HOG1* introns in PH-1 and Δ*lsm8* under untreated and osmotic stress (1 M KCl). **F** Western blot showing total Hog1 protein levels in PH-1 and Δ*lsm8*. Numbers indicate relative protein abundance normalized to PH-1. Tubulin is a loading control. **G** Western blot showing Hog1 phosphorylation (p-Hog1) levels in PH-1 and Δ*lsm8* under unstressed and 1 M KCl-treated conditions. Total Hog1 is a loading control. **H** Intracellular glycerol content in PH-1 and Δ*lsm8* under normal conditions. Data showing in (**B**), (**D**), (**E**) and (**H**) are mean ± SD, *n* = 3. Different letters indicate a significant difference (*P* < 0.05) based on one-way ANOVA followed by Tukey’s HSD test. and unpaired Student’s *t*-test

*F. graminearum* encounters diverse environmental stresses during host infection and survival. Given the critical roles of mitogen-activated protein kinase (MAPK) cascades in mediating stress responses in this fungus, including those to osmotic, oxidative, and cell wall stresses (Yun et al. 2014; Zheng et al. 2012; Gu et al. 2015; Ren et al. 2022), and the previously observed impact of *LSM8* deletion on these key signaling pathways, we hypothesized that Δ*lsm8* would compromise stress adaptation. To test this, we assessed the sensitivity of Δ*lsm8* to a panel of eight stress agents, including osmotic stressors (1 M NaCl, 1 M KCl, 0.5 M CaCl_2_), oxidative stress (0.05% H_2_O_2_), cell membrane stress (0.02% SDS), cell wall stress (200 μg/mL Congo red), and fungicides (0.25 μg/mL tebuconazole; 0.025 μg/mL fludioxonil). The Δ*lsm8* displayed heightened sensitivity to all tested conditions, with particularly pronounced hypersensitivity to the osmotic stressors and the fungicide fludioxonil (Fig. 4A, B).

Given the central role of the high osmolarity glycerol (HOG)-MAPK cascade in the osmoadaptation of filamentous fungi, we focused our investigation on Hog1, a core kinase within this pathway crucial for osmotic stress responses (Zheng et al. 2012; Zhang et al. 2023). Strand-specific RNA-seq data and Integrative Genomics Viewer (IGV) visualization revealed that *HOG1* possesses eight introns and exhibited obvious IR in Δ*lsm8*, indicative of substantially reduced splicing efficiency (Fig. 4C). While RT-qPCR analysis confirmed that total *HOG1* mRNA abundance remained unchanged under both unstressed and KCl treated conditions (Fig. 4D), IR analysis demonstrated markedly decreased splicing efficiency across nearly all eight introns in Δ*lsm8*, with these defects becoming more pronounced under stress conditions (Fig. 4E). Consistent with these splicing defects, immunoblotting further confirmed a significant reduction in total Hog1 protein levels in Δ*lsm8* compared to the wild type (Fig. 4F). These findings collectively establish that Δ*lsm8* does not directly regulate *HOG1* gene transcription, but is indispensably required for accurate *HOG1* pre-mRNA splicing to ensure proper protein synthesis and sustain robust osmotic stress responses. Unexpectedly, despite the significantly reduced total Hog1 protein levels in Δ*lsm8*, phospho-immunoblotting revealed remarkably elevated levels of phosphorylated Hog1 (p-Hog1) under both basal and osmotic stress conditions (Fig. 4G). This observation is consistent with potential hyperactivation of residual Hog1 protein and/or altered pathway feedback mechanisms. In agreement with a hyperactivated HOG response, intracellular glycerol accumulation was approximately three-fold higher in Δ*lsm8* than in PH-1 (Fig. 4H). Furthermore, we observed conspicuous splicing abnormalities in other critical nodes of the HOG-MAPK signaling pathway, including the downstream transcription factor *ATF1* (Van Nguyen et al. 2013; Jiang et al. 2015), in Δ*lsm8* (Fig. S2). Taken together, these data demonstrate that Lsm8 plays a critical role in enforcing the splicing fidelity of key components within the HOG-MAPK signaling pathway. By ensuring the proper production of functional proteins like Hog1, Lsm8 thereby supports osmoadaptation and broader environmental stress tolerance in *F. graminearum*.

### Lsm8 is required for asexual and sexual development

Asexual conidia and sexual ascospores, produced within perithecia, are pivotal for the infection cycle and overwintering survival of *F. graminearum*, contributing significantly to the devastating FHB disease. Our previous transcriptomic analysis revealed that the *LSM8* gene is actively expressed during crucial reproductive stages, including conidiation, conidial germination, and sexual development.

**Fig. 5:**
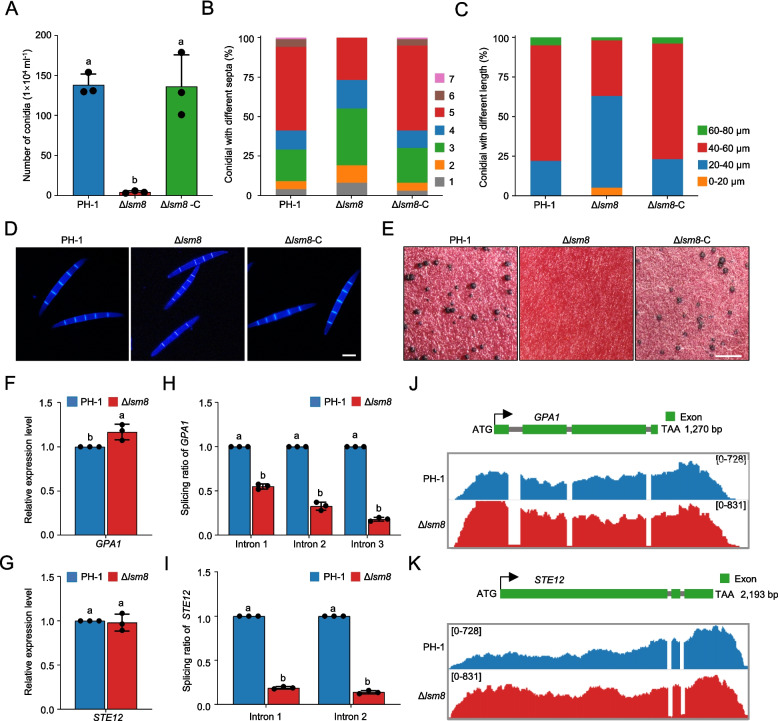
Lsm8 is essential for asexual and sexual reproduction by modulating pre-mRNA splicing of key developmental regulators. **A** Quantitative analysis of conidial production by PH-1, Δ*lsm8*, and Δ*lsm8*-C grown in CMC medium for 3 days. **B** Distribution of conidia with different septation numbers in PH-1, Δ*lsm8* and Δ*lsm8*-C. **C** Measurement of conidial lengths for PH-1, Δ*lsm8* and Δ*lsm8*-C. **D** Representative images of conidial morphology from PH-1, Δ*lsm8* and Δ*lsm8*-C. Scale bar = 10 μm. **E** Perithecium formation on carrot agar medium after 14 days of incubation under black light. Representative images show perithecia formed by PH-1, Δ*lsm8* and Δ*lsm8*-C. Scale bar = 1 mm. **F**, **G** Relative expression levels of *GPA1* and *STE12* pre-mRNAs in PH-1 and Δ*lsm8* determined by RT-qPCR. **H**, **I** Quantification of intron retention rates for *GPA1* (three introns) and *STE12* (two introns) in PH-1 and Δ*lsm8*. **J**,** K** Integrative Genomics Viewer (IGV) visualization of RNA-seq data illustrating altered intron retention patterns for *GPA1* and *STE12* in Δ*lsm8* compared to PH-1. Data in (**A**-**C**), (**F**-**I**) are presented as means ± SD from three independent biological replicates. Different letters indicate a significant difference (*P* < 0.05) based on one-way ANOVA followed by Tukey’s HSD test. and unpaired Student’s *t*-test

To elucidate its functional significance, we investigated the reproductive phenotypes of Δ*lsm8*. Compared to PH-1, Δ*lsm8* produced significantly fewer conidia in CMC broth and exhibited aberrant conidial morphology (reduced septation and shorter length) (Fig. 5A-D). Furthermore, Δ*lsm8* completely lost the ability to form perithecia on carrot agar medium, signifying a complete abolishment of sexual reproduction (Fig. 5E). These severe defects in asexual and sexual reproduction were fully restored in the complemented strain Δ*lsm8*-C (Fig. 5A-E). To unravel the molecular mechanism underlying the role of Lsm8 in fungal reproductive development, we conducted an in-depth analysis of genes exhibiting significant IR in Δ*lsm8*, specifically focusing on those previously implicated in fungal reproduction. Our comprehensive analysis identified two key developmental regulators Gpa1 and Ste12. *GPA1* encodes the alpha subunit of a heterotrimeric G protein, a central component of signal transduction pathways known to govern both asexual and sexual development in *F. graminearum*. Similarly, Ste12 is a critical transcription factor essential for sexual reproduction (Yu et al. 2008; McCarthy et al. 2012). Although RT-qPCR analysis revealed no significant or minor changes in the overall transcriptional levels of *GPA1* (Fig. 5F) or *STE12* (Fig. 5G) in Δ*lsm8* compared to PH-1, intron-retention analyses demonstrated a marked reduction in their pre-mRNA splicing ratio (Fig. 5H, I). IGV visualization further confirmed these findings, showing a substantial increase in IR rates for all three introns of *GPA1* in Δ*lsm8* (increases of 0.08, 0.06, and 0.28, respectively) (Fig. 5J). Likewise, both introns of *STE12* exhibited elevated retention rates (increases of 0.11 and 0.07, respectively) in the mutant (Fig. 5K; Tables S3-4). These data collectively suggest that Lsm8 regulates the processes of asexual and sexual reproduction in *F. graminearum* by ensuring the accurate pre-mRNA splicing of critical developmental regulators, including Gpa1 and Ste12.

### Lsm8 is crucial for full virulence and DON biosynthesis

**Fig. 6:**
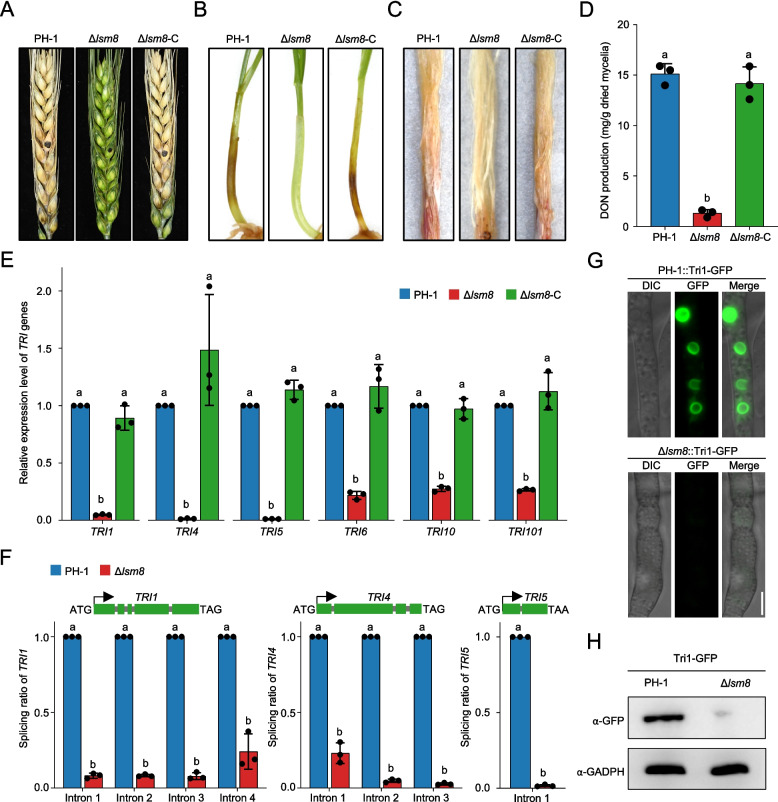
Lsm8 regulates virulence and DON biosynthesis through control of *TRI* gene pre-mRNA splicing. **A**−**C** Disease symptoms caused by indicated strains. Representative images illustrating disease severity infected by PH-1, Δ*lsm8*, and Δ*lsm8*-C on (**A**) wheat heads, (**B**) wheat coleoptiles, and (**C**) corn silks. **D** Quantification of DON production in cultures of PH-1, Δ*lsm8*, and Δ*lsm8*-C. **E** Relative mRNA expression levels of key *TRI* genes. Transcript levels of *TRI1*, *TRI4*, *TRI5*, *TRI6*, *TRI10*, and *TRI101* in the indicated strains, determined by RT-qPCR. **F** Splicing efficiency of *TRI* gene transcripts. Splicing ratios for *TRI1*, *TRI4*, and *TRI5* transcripts in PH-1 and Δ*lsm8*, assessed by RT-qPCR. Statistical significance was determined by one-way ANOVA followed by Tukey’s HSD test. and unpaired Student’s t-test; different letters above bars indicate a significant difference (*P* < 0.05). **G** Impaired DON toxisome formation in Δ*lsm8*. Confocal microscopy images showing Tri1-GFP indicated toxisome formation in PH-1 and Δ*lsm8* grown in TBI medium for 2 days. Scale bar = 5 µm. **H** Western blot analysis of Tri1-GFP protein levels. Immunoblot detecting Tri1-GFP fusion protein levels in PH-1 and Δ*lsm8*. GAPDH served as an internal loading control

*F. graminearum* produces the mycotoxin deoxynivalenol (DON), a key virulence factor, via the trichothecene biosynthetic gene (*TRI*) cluster, including key genes such as *TRI5*, *TRI6*, and *TRI10* (Alexander et al. 2009). To define the role of Lsm8 in pathogenicity and DON biosynthesis, we performed infection assays on various host tissues and quantified DON production. Δ*lsm8* was nearly nonpathogenic compared with PH-1 (Fig. 6A-C). Further analysis showed that DON production was significantly reduced in Δ*lsm8* (Fig. 6D). The Δ*lsm8*-C restored both pathogenicity and toxin production to wild-type levels. These results suggest that Lsm8 influences virulence by regulating DON biosynthesis.

To unravel the molecular mechanisms by which Lsm8 regulates pathogenicity and DON biosynthesis, we investigated its impact on the expression of *TRI* cluster genes. Transcriptomic analysis revealed a significant downregulation of multiple key *TRI* genes in Δ*lsm8*, including *TRI1, TRI4, TRI5, TRI6, TRI10*, and *TRI101* (Fig. 6E). Moreover, compared with the wild type, splicing efficiency of *TRI1, TRI4,* and *TRI5* was significantly reduced in Δ*lsm8* (Fig. 6F), indicating that Lsm8 plays an important role in proper splicing of *TRI* mRNAs. The abnormal splicing ultimately leads to a decrease in the content of correctly translated proteins. We then investigated DON toxisome formation, a specialized subcellular compartment for DON biosynthesis formed through endoplasmic reticulum remodeling (Menke et al. 2013; Tang et al. 2018). To compare toxisome formation in wild-type and Δ*lsm8* backgrounds, we targeted the DON toxisome indicator protein Tri1-GFP. Results showed that in the wild-type background, Tri1-GFP aggregated to form typical spherical toxisomes, whereas almost no toxisomes were observed in Δ*lsm8* (Fig. 6G). Western blot experiments also showed that the content of Tri1-GFP fusion protein was dramatically reduced in Δ*lsm8* (Fig. 6H). Collectively, these findings indicate that Lsm8 regulates *TRI* gene expression both transcriptionally and post-transcriptionally via splicing, ultimately controlling DON synthesis and full virulence in *F. graminearum*.

### Predominant role of RNA exosome in degrading intron-retained transcripts in Δ*lsm8*

**Fig. 7:**
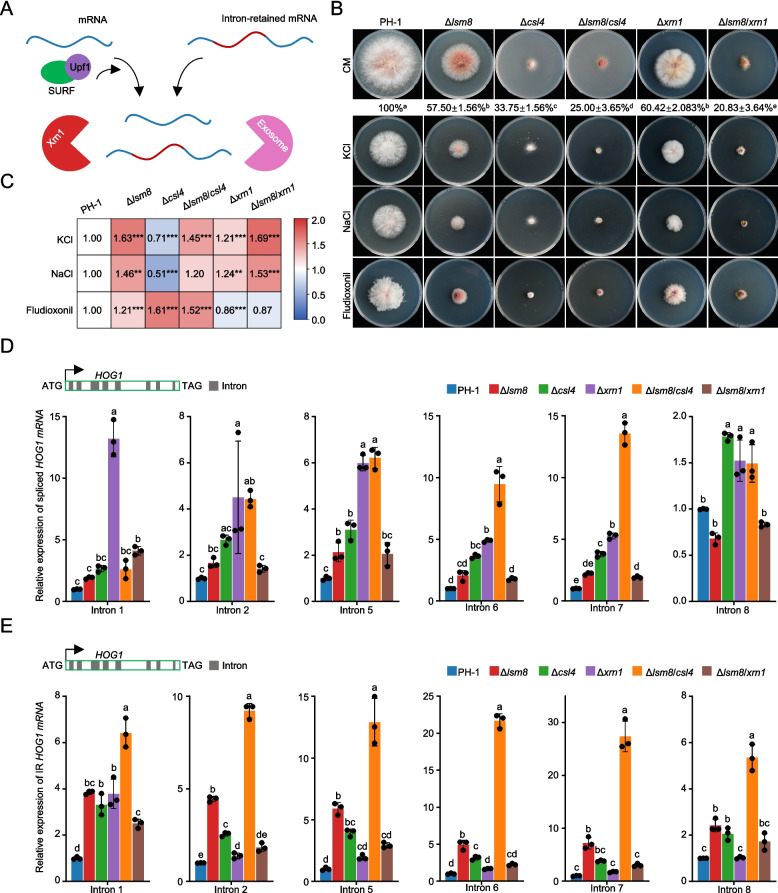
RNA exosome complex is the primary degrader of intron-retained transcripts in Δ*lsm8*. **A** Schematic representation of mRNA decay pathways, illustrating the general mechanisms for normal mRNA and intron-retained mRNA degradation, highlighting key exonucleases involved. **B** Phenotypic analysis of vegetative growth and stress responses in various strains. Relative growth rates of mutant strains compared to PH-1 are indicated below the respective growth phenotypes. **C** Quantification of growth inhibition rates for PH-1 and mutant strains exposed to osmotic stress agents. The sensitivity of PH-1 was normalized to 1.0 for comparative analysis. Statistical significance is denoted by asterisks: *P < 0.05, **P < 0.01, ***P < 0.001 (one-way ANOVA followed by unpaired Student’s *t*-test). **D** Relative expression levels of spliced *HOG1* mRNA transcripts in PH-1 and various mutant strains. **E** Relative expression levels of intron-retained *HOG1* mRNA transcripts in PH-1 and various mutant strains. Data in (**B**), (**D**), and (**E**) represent mean ± SD from three independent biological replicates (*n* = 3). Different letters above bars indicate statistically significant differences (*P* < 0.05) determined by one-way ANOVA followed by Tukey’s HSD (HSD) test

In eukaryotes, aberrant transcripts, particularly those exhibiting IR, are primarily recognized, and degraded through the nonsense-mediated mRNA decay (NMD) pathway. This crucial mechanism prevents the accumulation of potentially toxic truncated proteins by activating the UPF1–SMG1–eRF1/3 (SURF) complex, centered around Upf1 (Isken et al. 2008). This complex directly inhibits translation of aberrant transcripts and recruits key mRNA degradation enzymes, including the 5′−3′ exonuclease Xrn1 and the RNA exosome complex (Fig. 7A). To delineate the specific exoribonucleases involved in the degradation of IR transcripts in *F. graminearum*, we performed sequence homology analyses using known yeast exonucleases as references. This led to the identification of orthologs encoding Csl4, a subunit of the 3′−5′ exosome complex, and Xrn1 in this fungus.

To investigate their roles, we generated single deletion mutants (Δ*csl4*, Δ*xrn1*) and double mutants (Δ*lsm8*/*xrn1*, Δ*lsm8*/*csl4*). Initial growth phenotyping assays revealed that both Δ*csl4* and Δ*xrn1* exhibited significant growth defects. These defects were further exacerbated in the Δ*lsm8*/*xrn1* and Δ*lsm8*/*csl4* when compared to Δ*lsm8* (Fig. 7B). Building upon our previous findings that Lsm8 regulates osmotic stress adaptation by controlling *HOG1* pre-mRNA splicing (Fig. 4), we assessed the sensitivity of these mutants to three osmotic stressors. Both Δ*csl4* and Δ*xrn1* displayed significantly altered sensitivities to tested osmotic stress agents compared to PH-1, though notably, they presented contrasting trends. The osmotic phenotypes of the double mutants more closely resembled that of Δ*lsm8* (Fig. 7C). These findings implied that both Xrn1 and Csl4 might involve in regulating aberrant mRNA accumulation in Δ*lsm8*.

To further elucidate the contributions of Xrn1 and Csl4 to IR transcript degradation, we initially focused on *HOG1* pre-mRNA, a well-established Lsm8 substrate (Fig. 4). We quantified the relative expression levels of mature *HOG1* mRNA and its intron-retained transcripts (specifically assessing retention in introns 1, 2, 5, 6, 7 and 8). Our results showed a significant increase in mature *HOG1* mRNA transcripts in both Δ*xrn1* and Δ*csl4* compared to PH-1 (Fig. 7D). Meanwhile, IR transcripts markedly accumulated in Δ*csl4*, with an even more pronounced increase observed in the Δ*lsm8*/*csl4*. While Δ*xrn1* also exhibited elevated levels of IR transcripts compared to PH-1, this accumulation was significantly lower than that observed in Δ*csl4* (Fig. 7E). Collectively, these results demonstrate that the exosome complex plays a major and predominant role in degrading intron-retained transcripts that accumulate due to Lsm2-8 complex dysfunction, while Xrn1 contributes to this process to a comparatively lesser extent in *F. graminearum*.

## Discussion

RNA-binding proteins (RBPs) are central orchestrators of co- and post-transcriptional gene regulation in eukaryotes, with many functioning as critical splicing factors. Lsm8, a canonical RBP, facilitates splicing by binding to the 3′ end of RNA. Such splicing-associated RBPs are known to modulate multiple layers of gene expression and are crucial for adaptive responses to abiotic stresses (Muthusamy et al. 2021). For instance, in *F. graminearum*, deletion of the splicing factor Sgh1, an RBP, confers hypersensitivity to osmotic and cell wall stresses, while loss of the RBP Nam8 increases susceptibility to calcium stress (Wang et al. 2022; Jiang et al. 2025). However, the precise mechanisms by which these splicing factors contribute to stress responses have remained largely unknown. Our findings establish a direct link between RNA splicing and fungal stress signaling. We observed that the stress response profile of Δ*lsm8* closely phenocopies that of a Δ*hog1* mutant, a central component of the HOG-MAPK signaling pathway. Further investigations revealed that Lsm8 directly regulates the splicing efficiency of both *HOG1* and *ATF1*, thereby modulating the phosphorylation status of Hog1. This study thus identifies Lsm8 as a key coordinator of spliceosome function and MAPK-mediated stress adaptation, providing a novel perspective on the integration of RNA processing and stress signaling in fungi.

Mycotoxins, as secondary metabolites, represent a significant global threat to food security and human and animal health. In *F. graminearum*, DON biosynthesis is governed by the *TRI* gene cluster, whose regulation spans multiple levels from transcription to translation. Transcriptional control involves direct regulation by Tri10 and Tri6, complemented by the coordinated action of various transcription factors such as Pac1, CreA, AreA, and the VelB/VelA/LaeA complex, which respond to environmental cues like pH, carbon, nitrogen, and light (Merhej et al. 2011; Foroud et al. 2019; Villafana et al. 2019; Niu et al. 2024; Wang et al. 2025a, b). Beyond transcription, regulatory mechanisms extend to translation and protein stability. For example, deletion of *FgSfp1* enhances *TRI* expression but paradoxically decreases the protein levels of *TRI* genes by interacting with the RNA 2′-O-methyltransferase FgNop1, thereby influencing mRNA translation rates (Sun et al. 2024). Additionally, Ubp15 regulates Tri4 deubiquitination for protein stabilization, and Isw1 modulates TRI protein translation by affecting tRNA expression without altering transcription (Chen et al. 2024; Wang et al. 2025a, b). Epigenetic regulation also plays a critical role, with histone modifications such as histone acetylation, H3K4me2/3 methylation, and H2B ubiquitination promoting *TRI* gene expression (Chen et al. 2018; Ma et al. 2021), while H3K27me3 deposition exerts a repressive effect (Tang et al. 2021). Post-transcriptional control includes the suppression of antisense-*TRI5* and long non-coding RNAs (RNA5P) by Tri10 and Tri6, both of which negatively regulate DON biosynthesis (Huang et al. 2024). Notably, regulatory mechanisms at the pre-mRNA splicing level have remained largely unexplored. Our study reveals that Lsm8 is essential not only for maintaining the basal expression of *TRI* genes but also for ensuring the precise splicing of a subset of *TRI* transcripts. This finding unveils a novel layer of post-transcriptional control in DON biosynthesis, expanding our understanding of the complex regulatory network governing this critical virulence factor.

The significance of Lsm8's role in *F. graminearum* is further highlighted by comparing its function with other characterized splicing factors. Core splicing factors, such as the RNA recognition motif (RRM) protein FgRbp1, whose deletion causes intron retention in 4,849 genes (Wang et al. 2021), and the kinase FgPrp4, which affects over 60% of genes (Gao et al. 2016), play pivotal and broad roles in splicing, leading to widespread intron retention upon their deletion. Consistent with these, Lsm8, as a core component of the U6 snRNP, also plays a foundational role, with Δ*lsm8* resulting in significant intron retention in 1,821 genes, thus positioning it as a critical general splicing factor. In contrast, other factors such as SR proteins (e.g., FgSrp1) exhibit more limited and specific effects on splicing patterns: FgSrp1 deletion mutant affects 139 genes and reduces both asexual development and pathogenicity (Zhang et al. 2017); FgSrp2 influences 130 AS events with minimal growth or virulence defects (Zhang et al. 2020); FgSrk1 alters 74 AS events across 62 genes, 60% of which are IR (Wang et al. 2018); and the SR-like protein Sgh1 impacts 325 AS events, including 216 IRs (Wang et al. 2022). Critically, our study reveals a unique distinguishing feature of Lsm8 loss: the global and active compensatory transcriptional feedback of numerous splicing factor genes (including intronless ones) in Δ*lsm8* (Fig. S3). This mechanism, triggered by widespread splicing impairment, represents a secondary regulatory response that has not been previously reported for the other characterized splicing factors in *F. graminearum*. These observations thus suggest that Lsm8 is a core splicing factor whose loss initiates widespread splicing defects, which are then followed by this active compensatory feedback upregulation of spliceosomal component expression.

The widespread IR observed upon Lsm8 deficiency often triggers diverse RNA surveillance pathways that determine the fate of IR transcripts. In the nucleus, IR transcripts can either be degraded or undergo delayed splicing under specific conditions. In the cytoplasm, they may be degraded by NMD, cleaved by miRNAs, or even translated into truncated or novel protein isoforms (Jacob and Smith 2017). In S. cerevisiae, NMD is partially coupled with splicing, where the NMD factor Upf1 assists in removing aberrant IR transcripts (Kawashima et al. 2009). Similarly, in mice, the NMD factor UPF2 significantly reduces alternative splicing transcripts, with approximately half of the retained introns harboring premature termination codons (PTCs) (Weischenfeldt et al. 2012). However, IR transcripts do not universally adhere to the classical NMD model. In A. thaliana, for instance, most IR transcripts, despite containing PTCs, are resistant to NMD, whereas other AS types, such as exon skipping, are more efficiently eliminated (Kalyna et al. 2011). Consistent with this, the NMD pathway in *F. graminearum* does not appear to alter the abundance of PTC-containing or truncated transcripts (Lu et al. 2022).

In eukaryotes, the RNA exosome mediates 3′ → 5′ mRNA decay and Xrn1/Xrn2 mediate 5′ → 3′ mRNA decay. Typically, Xrn2 localizes to the nucleus, whereas the RNA exosome and Xrn1 are distributed across both the nucleus and cytoplasm. Nuclear exosome and Xrn2 have been reported to preferentially target IR transcripts (Fig. S4) (Kilchert et al. 2016; Davidson et al. 2012). Our results reveal a functional coupling between Lsm8-mediated RNA splicing and the RNA exosome. First, AC-MS analysis demonstrated that Lsm8 physically interacts with core exosome subunits, including Rrp45, Rrp4, and Dis3. Second, Δ*lsm8* activated RNA surveillance and degradation pathways, notably leading to a compensatory upregulation of the RNA exosome, which is likely responsible for clearing these accumulated IR transcripts resulting from Lsm8 deficiency. This is robustly supported by the substantial and further accumulation of IR transcripts observed in the Δ*lsm8*/*csl4* (Lsm8-exosome double mutant). This differential contribution suggests a distinct division of role within the RNA surveillance pathway. The exosome, functioning as a major 3′−5′ exoribonuclease complex in both the nucleus and cytoplasm, is well-positioned to handle aberrantly spliced, nuclear-retained IR transcripts. Xrn1, conversely, is primarily a 5′−3′ exoribonuclease predominantly active in the cytoplasm, generally targeting decapped mRNAs (Fig. S4). Therefore, it is plausible that the exosome efficiently clears nuclear or nascent IR transcripts, while Xrn1's role becomes more prominent for normally spliced, decapped mRNAs or possibly for IR transcripts that manage to exit the nucleus. Regarding the 5′ → 3′ RNA decay pathway, Δ*lsm8* showed a significant induction of Xrn2 (log_2_FC = 1.18, *P* < 0.05), while Xrn1 just mildly induced (log_2_FC = 0.71, *P* < 0.05), implying distinct roles in response to widespread splicing defects. Given the severe growth defect of the Δ*xrn2*, the Δ*lsm8*/*xrn2* was not constructed. Genetic interaction analysis revealed a strong synergistic defect in Δ*lsm8*/*xrn1*, suggesting a synergistic role for Lsm8 and Xrn1 in RNA surveillance. In contrast, the defect in Δ*lsm8*/*csl4* was weaker than the simple additive effect, indicating partial functional redundancy and a buffering effect between Lsm8 and RNA exosome. Notably, in *F. graminearum* the location of RNA exosome (Yuan et al. 2023) and Xrn1/2 are similar to model organism (Fig. S4). These results suggest that IR transcripts are likely preferentially degraded in the nucleus before being exported to the cytoplasm. Collectively, these findings establish Lsm8 as a pivotal factor in coordinating RNA splicing with RNA surveillance pathways, ensuring splicing fidelity and regulating gene expression in *F. graminearum*.

The strong conservation of both the Lsm2-8 complex (including Lsm8) and the RNA exosome across eukaryotes, including diverse fungal species, suggests that the intricate coordination between Lsm8-mediated splicing and exosome-mediated RNA surveillance represents a fundamental and conserved mechanism essential for fungal viability and virulence. For instance, in the human fungal pathogen *Candida albicans*, deletion of Lsm proteins impairs filamentation, thereby further compromising its pathogenicity (Lash et al. 2024). Therefore, targeting such a core, conserved module that orchestrates fundamental processes like stress adaptation and mycotoxin biosynthesis (as shown in *F. graminearum*) could offer a promising strategy for developing novel, broad-spectrum antifungal agents. This would potentially disrupt vital fungal biology across a range of pathogens, thus enhancing the broader translational impact and application prospects of our findings beyond *F. graminearum*. Overall, this study establishes Lsm8 as a central regulator linking RNA splicing to RNA surveillance and offers a framework for investigating the broader biological and antifungal significance of the Lsm8-exosome module in pathogenic fungi.

## Conclusion

This study demonstrates that Lsm8, a deeply conserved core subunit of the eukaryotic Lsm2-8 complex, functions as an essential component within a conserved Lsm8–exosome module that critically maintains RNA splicing fidelity in *F. graminearum*. Disruption of *LSM8* profoundly perturbs fungal biology, severely impairing growth, development, environmental stress adaptation, virulence, and deoxynivalenol (DON) biosynthesis. We delineate a mechanistic link between Lsm8-mediated splicing and stress adaptation through its regulation of *HOG1* and *ATF1* splicing efficiency and modulation of Hog1 phosphorylation. Moreover, Lsm8 establishes a post-transcriptional layer of control over mycotoxin production by ensuring accurate splicing of key *TRI* transcripts required for DON biosynthesis. Mechanistically, *LSM8* deletion leads to the accumulation of intron-retained transcripts that are preferentially degraded by the RNA exosome, revealing a conserved Lsm8–exosome module that couples RNA splicing fidelity with RNA surveillance. Collectively, these findings define a fundamental post-transcriptional regulatory mechanism governing fungal virulence and mycotoxin biosynthesis, highlighting RNA-processing factors as universal determinants of pathogenicity. Given the deep evolutionary conservation of this module across eukaryotes, it represents a promising broad-spectrum target for developing innovative strategies to control fungal diseases and mitigate mycotoxin contamination, thereby contributing to global food security.

## Methods and materials

### Strains, culture conditions, and plant material

*F. graminearum* strain PH-1 (NRRL 31084)*,* originally isolated from corn in Michigan, was provided by the Mycology Laboratory of the Zhejiang University (Hangzhou, China). The FHB-susceptible wheat (*Triticum aestivum* L.) cultivar "Jimai-22" (developed through hybridization by the Crop Research Institute of Shandong Academy of Agricultural Sciences, China) was used to test the fungal pathogenicity. To evaluate growth phenotypes, fungal strains were inoculated on potato dextrose agar (PDA; 200 g potato, 20 g glucose, 10 g agar per liter), complete medium (CM; 10 g of glucose, 2 g peptone, 1 g yeast extract, 1 g casamino acids, 6 g NaNO_3_, 0.52 g KCl, 0.52 g MgSO_4_·7H_2_O, 1.52 g KH_2_PO_4_, 0.01% trace elements, 0.01% of vitamins, 1% agar per liter, pH 6.5), minimal medium (MM; 2 g NaNO_3_, 0.5 g KCl, 0.5 g MgSO_4_·7H_2_O, 1 g KH_2_PO_4_, 0.01 g FeSO_4_·7H_2_O, 30 g Sucrose, 0.005% trace element and 1% agar per liter, pH 7.0), and yeast extract peptone dextrose agar (YEPD; 3 g yeast extract, 10 g peptone, 20 g glucose, 1% agar per liter, pH 6.5), and incubated at 25 °C for 3 days. Colony diameters were measured to assess growth rate. For stress assays, strains were inoculated onto CM plates supplemented with different stress agents: NaCl (1 M), KCl (1 M), CaCl_2_ (0.5 M), H_2_O_2_ (0.05%), SDS (0.02%), Congo Red (200 μg/mL), Tebuconazole (0.25 μg/mL), Fludioxonil (0.025 μg/mL). All experiments were independently repeated three times.

### Construction of gene deletion and complementation strains

The Δ*lsm8*, Δ*csl4*, Δ*csl4*/*lsm8*, Δ*xrn1*, Δ*xrn1*/*lsm8* gene deletion assays via homologous recombination using the double-joint PCR method (Yu et al. 2004). Flanking sequences upstream and downstream of the target gene were amplified using primers listed in Table S1 and flanking sequences of *LSM8* fused with the hygromycin resistance cassette (*HPH*) and flanking sequences of Csl4 and Xrn1 were used with the geneticin (*G418*). The fusion fragment was introduced into the PH-1 strain or Δ*lsm8* by polyethylene glycol (PEG)-mediated protoplast transformation and selected on medium containing 100 μg/mL hygromycin or 100 μg/mL geneticin. Putative deletion transformants, in which the ORF was replaced by the *HPH*/*G418* cassette, were verified by PCR. For complementation, the ORF of *LSM8* was amplified using primers listed in Table S1 and co-transformed with a linearized pYF11-GFP vector digested by XhoI into the yeast strain XK1-2554 using the Alkali-Cation Yeast Transformation Kit (112200200, MP Biomedicals, USA), followed by selection on SD-Leu medium (26.7 g Minimal SD base, 0.74 g -Trp Do supplement, 1% agar per liter, pH 6.7) at 30 °C for 3 days. Positive recombinants were verified and plasmids were extracted by using the yeast plasmid extract kit (D1160, Solarbio, China). The recombinant plasmid transferred into *E. coli* DH5α to propagation. The verified plasmid was then introduced into the Δ*lsm8* deletion mutant via PEG-mediated protoplast transformation and selected on geneticin G418 to obtain ectopic complementation or tagged fusion strains.

### Yeast two-hybrid (Y2H) assay

The full length ORFs of Lsm2/3/4/8 were amplified from PH-1 cDNA and cloned into bait (pGBKT7) and prey (pGADT7) vectors (Clontech) via ClonExpress II One Step Cloning Kit (C112-01, Vazyme, China) with primers in Table S1, respectively. After verification, bait vector and each prey vector were co-transformed into *S. cerevisiae* Y2H Gold following the LiAc/ss-DNA/PEG (lithium acetate/single-stranded DNA/polyethylene glycol) transformation protocol. Positive and negative controls were pGBKT7-53/pGADT7-T and pGBKT7-Lam/pGADT7-T, respectively. Transformants were selected on SD -Leu/-Trp (26.7 g Minimal SD base, 0.64 g -Leu/-Trp DO supplement, 1% agar per liter, pH 6.7) plates at 30 °C for 3 days, then plated on SD -Leu/-Trp/-His/-Ade (26.7 g Minimal SD base, 0.62 g -Leu/-Trp DO supplement, 1% agar per liter, pH 6.7) plates for interaction assays after 4 days (Tang et al. 2021). Experiments were performed in triplicate.

### Bimolecular fluorescence complementation (BiFC) assay

The ORF of Lsm4 was cloned into the NYFP vector (hygromycin resistant), and the ORF of Lsm8 was cloned into the CYFP vector (zeocin resistant) within pHZ65 using primers in Table S1. Verified constructs were co-transformed into PH-1 strain and selected on media containing both antibiotics. The selected strains were incubated in YEPD media 24 h at 25 °C with shaking at 180 rpm. Fluorescence signals were observed using a Zeiss LSM880 confocal microscope.

### Affinity capture-mass spectrometry (AC-MS)

GFP-tagged Lsm8 constructs were introduced into the Δ*lsm8* strain. Approximately 2 g of fresh mycelia were ground in liquid nitrogen and lysed in 1 mL of protein extraction buffer as described for Western blot assays. Lysates were incubated overnight at 4 °C with 40 µL of anti-GFP magnetic beads (SA070001, Smart-Life, China) under gentle rotation. Beads were washed five times with TBS buffer (20 mM Tris–HCl, 500 mM NaCl, pH 7.5). Bound proteins were eluted by boiling the beads in TBS containing 1–2% SDS for 10 min. After centrifugation, the supernatants were analyzed by mass spectrometry (QLbio, Beijing). Protein coverage was used to assess the relative abundance of specifically bound proteins.

### Co-immunoprecipitation (Co-IP) assay

Lsm2/3/4-GFP and Lsm8-Flag fusion constructs were co-transformed into the PH-1 strain and selected on media containing G418 and Hph (Hygromycin B). Protein extracts were prepared as described for Western blot assays, and 200 µL of each lysate was reserved as input. The remaining lysate was incubated with 25 µL of anti-GFP magnetic beads (SA070001, Smart-Life, China) for 6 h at 4 °C with rotation. Beads were washed five times with TBS buffer and boiled to elute immune complexes. Both IP and input samples were analyzed by Western blot using anti-Flag and anti-GFP antibodies. Anti-GAPDH was used as a loading control. Due to the small molecular weight of Lsm8-Flag,samples were transferred onto a 0.2 μm PVDF membrane (#1620177, Bio-Rad, USA). The protein is marked with a protein marker (RM19001, Abclonal, China). Two independent biological replicates were performed for each experiment.

### Perithecium formation and conidiation

PH-1, Δ*lsm8*, and Δ*lsm8*-C strains were cultured on carrot agar (CA; 200 g carrot, 1% agar per liter) plates at 25 °C under black light (wavelength, 365 nm, HKiv, Xiamen, China) for two weeks to induce perithecia formation. Carboxymethyl cellulose (CMC, 15 g carboxymethyl cellulose sodium, 1 g yeast extract, 1 g NH_4_NO_3_, 1 g KH_2_PO_4_, 0.5 g MgSO_4_·7H_2_O per liter, pH 7.0) liquid medium was used to induce conidiation. Fresh 10 mm hyphal plugs were taken from the colony edges of wild-type, Δ*lsm8*, and Δ*lsm8*-C strains and inoculated into CMC medium. Cultures were incubated at 25 °C with shaking at 180 rpm for 5 days, after which conidia number and morphology were observed and quantified. Each experiment was repeated three times.

### Pathogenicity assays and DON measurement

Pathogenicity was assessed through a multi-faceted approach using the susceptible wheat cultivar Jimai 22 for relevant assays. Conidial suspension (1 × 10^5^ conidia/mL) was inoculated into the central spikelets of flowering wheat heads. Infected spikelets were subsequently counted after 14 days, with 10 replicates performed per strain. Additionally, 5 mm mycelial plugs were inoculated onto wheat coleoptiles, which were then incubated at 25 °C for 3–5 days before lesion lengths were measured. Parallel to these wheat assays, 5 mm mycelial plugs were also inoculated into maize silks under identical incubation conditions. Each assay was repeated three times. For DON quantification, strains were cultured in trichothecene biosynthesis induction broth (TBI; 30 g sucrose, 1 g KH_2_PO_4_, 0.5 g MgSO_4_·7H_2_O, 0.5 g KCl, 0.01 g FeSO_4_·7H_2_O, 1.47 g putrescine hydrochloride, 0.05% trace element per liter, pH 4.5) medium with shaking at 180 rpm, 28 °C in the dark for 7 days. DON in the culture supernatants were quantified using a commercial ELISA kit (Wis008, Wise Science, China).

### RNA-seq and RT-qPCR analysis

PH-1 and Δ*lsm8* strains were cultured in YEPD liquid medium at 25 °C for 24 h with 180 rpm, three biological replicates per group. Library construction and sequencing were performed by Novogene using the Illumina HiSeq2000 platform. Low-quality reads and adapter sequences were trimmed using Trimmomatic v0.39 (Bolger et al. 2014). Clean reads were aligned to the reference genome using HISAT2 v2.2.1 (Gill and Dhillon 2022). Gene read counts were quantified with featureCounts v1.6.0 (Liao et al. 2014). Differential expression analysis was conducted using edgeR v3.36.0 and limma v3.50.0 (McCarthy et al. 2012; Ritchie et al. 2015), with thresholds of |log_2_FC|> 1 and *P* < 0.05. KEGG pathway enrichment was analyzed via the DAVID database (https://david.ncifcrf.gov/). For RT-qPCR, fresh mycelia were harvested from strains grown in YEPD, CM, or TBI liquid media. RNA was extracted using TaKaRa RNAiso reagent and reverse-transcribed with HiScript II Q RT Kit. Quantitative PCR was performed with SYBR qPCR Master Mix on a Bio-RAD real-time PCR system. Primers are listed in Table S1. ACTIN served as the internal control, and relative expression was calculated using the 2^–ΔΔCt^ method.

### Western blot assays

Strains were cultured in YEPD or TBI liquid media for harvesting fresh mycelia. Mycelia were ground in liquid nitrogen and lysed in protein extraction buffer (50 mM Tris–HCl pH 7.5, 150 mM NaCl, 5 mM EDTA, 1% Triton X-100, 1:100 protease inhibitor cocktail) on ice for 30 min. After centrifugation at 14,000 × g for 20 min, supernatants were collected, mixed with loading buffer, and boiled for 5 min. Samples were separated by 10% SDS-PAGE and transferred to PVDF membranes (IPVH00010, Millipore, Cork, Ireland). Membranes were probed with the following primary antibodies: anti-GFP (ab32146, Abcam, UK), anti-Flag (AE092, Abclonal, China), anti-Hog1 (Abclonal), and anti-p38 (#9212, Cell Signaling Technology, USA). The internal controls used were anti-Glyceraldehyde-3-Phosphate Dehydrogenase (GAPDH) (EM1101; HuaAn, China) or anti-Histone H3 (EM30605, HuaAn, China). Experiments were repeated twice.

### IR analysis and splicing efficiency measurement

IR events in *F. graminearum* were analyzed using IRFinder v1.3.0 (Middleton et al. 2017). Raw RNA-seq reads were quality controlled and trimmed with Trimmomatic v0.39, then aligned to the reference genome with HISAT2 v2.2.1 using IRFinder-recommended parameters. IRFinder builds an intron index from the reference genome FASTA and annotation GTF files, identifies testable introns, and excludes overlapping or low-complexity regions. IR ratio was calculated per intron as: IR ratio = (retained intron reads)/(retained intron reads + spliced reads). Differential IR analysis used IRFinder to built-in statistics with significance thresholds of |ΔIR ratio| > 0.1 and FDR-adjusted *P* < 0.05. Significantly different introns were annotated, and corresponding genes were subjected to KEGG enrichment analysis with q-value < 0.05 as cutoff. To validate splicing efficiency, total RNA was extracted from fresh mycelia, treated with DNase to remove genomic DNA, and reverse-transcribed. RT-qPCR was performed with three biological replicates. For spliced transcript quantification, the forward primer (F1) was designed to span the exon-exon junction (covering 10 bp upstream and 10 bp downstream of the intron boundary), ensuring amplification exclusively from fully spliced mRNA. The reverse primer R1 was located within the downstream exon (~ 150 bp from the splice junction). For intron-retained transcript quantification (F2), an intron-specific forward primer located within the intron region was used together with R1. This primer pair specifically amplifies unspliced transcripts retaining the intron (Fig. S5). Splicing efficiency (splicing ratio) was calculated as: 2^(Ct IR – Ct spliced)^, where the ratio reflects the relative abundance of spliced to unspliced transcripts at each site. Wild-type splicing efficiency was normalized to 1.

### Analysis of intracellular glycerol content

Intracellular glycerol concentration was measured using a glycerol assay kit (F005-2–1, Nanjing Jiancheng Bioengineering Institute, China) following the protocol. Strains were grown in CM for 12 h, harvested, washed, and homogenized in lysis buffer (10 μL per mg fresh weight). The supernatant was diluted 20-fold with ddH₂O, and absorbance at 550 nm was measured with a microplate reader to calculate glycerol content per unit fresh weight.

### Statistical analysis

All quantitative data are presented as mean ± standard deviation (SD) from at least three independent experiments. Statistical analyses were performed using GraphPad Prism version 10.0. Differences between two groups were assessed using the unpaired Student’s *t*-test. For comparisons among multiple groups, one-way analysis of variance (ANOVA) was conducted, followed by Tukey’s honestly significant difference (HSD) post hoc test. Statistical significance was defined at a threshold of *P* < 0.05. Differences among groups are indicated by distinct lowercase letters, with different letters denoting statistically significant differences. Alternatively, asterisks as follow *P < 0.05, **P < 0.01, ***P < 0.001.

## Supplementary Information

Supplementary Material 1: Figure S1. Molecular characterization of *F. graminearum* Lsm8 and validation of gene deletion and complementation strains.

Supplementary Material 2: Figure S2. Lsm8 regulates pre-mRNA splicing of *ATF1* in *F. graminearum*.

Supplementary Material 3: Figure S3. Intron retention and expression profiles of upregulated splicing factor and RNA degradation genes in WT and Δ*lsm8*.

Supplementary Material 4: Figure S4. Phenotypes and subcellular localization of Xrn1 and Xrn2 in *F. graminearum*.

Supplementary Material 5: Figure S5. RT-qPCR primer design for distinguishing spliced and intron-retained transcripts.

Supplementary Material 6: Table S1. List of primers used in this study.

Supplementary Material 7: Table S2. Identification of proteins interacting with Lsm8 via affinity purification-mass spectrometry.

Supplementary Material 8: Table S3. Differentially expressed genes in Δ*lsm8* compared to PH-1.

Supplementary Material 9: Table S4. Analysis of intron retention events in Δ*lsm8*.

## Data Availability

The RNA-seq analysis data that support the findings of this study are generated in this study have been deposited in the Genome Sequence Archive (GSA) hosted by the National Genomics Data Center (NGDC). The RNA-seq raw data, along with processed information, are available under the BioProject PRJCA046186. The source data of this study are available on Figshare with DOI number 10.6084/m9.figshare.30345082.

## Abbreviations

AC-MS: Affinity capture-mass spectrometry
AS: Alternative splicing
CO-IP: Co-immunoprecipitation
DON: Deoxynivalenol
FHB: Fusarium head blight
HOG: High osmolarity glycerol
MAPK: Mitogen-activated protein kinase
Lsm: Like Sm
IGV: Integrative Genomics Viewer
IR: Intron retention
NMD: Nonsense-mediated mRNA decay
RBP: RNA-binding protein
snRNA: Small nuclear RNA
SURF: SMG1–UPF1–release factors (eRF1/3) complex
*TRI*: Trichothecene biosynthetic genes
