# Supplementary material for: A conserved Lsm8–exosome module maintains RNA splicing fidelity to control fungal stress adaptation and virulence
Source: Stress Biol. 2026 Feb 10;6(1):14. doi: 10.1007/s44154-026-00285-6 (PMC12886710; doi:10.1007/s44154-026-00285-6)
Supplement: Supplementary file 1 — Supplementary Material 1: Figure S1. Molecular characterization of F. graminearum Lsm8 and validation of gene deletion and complementation strains. [file 44154_2026_285_MOESM1_ESM.pdf]

Figure S1

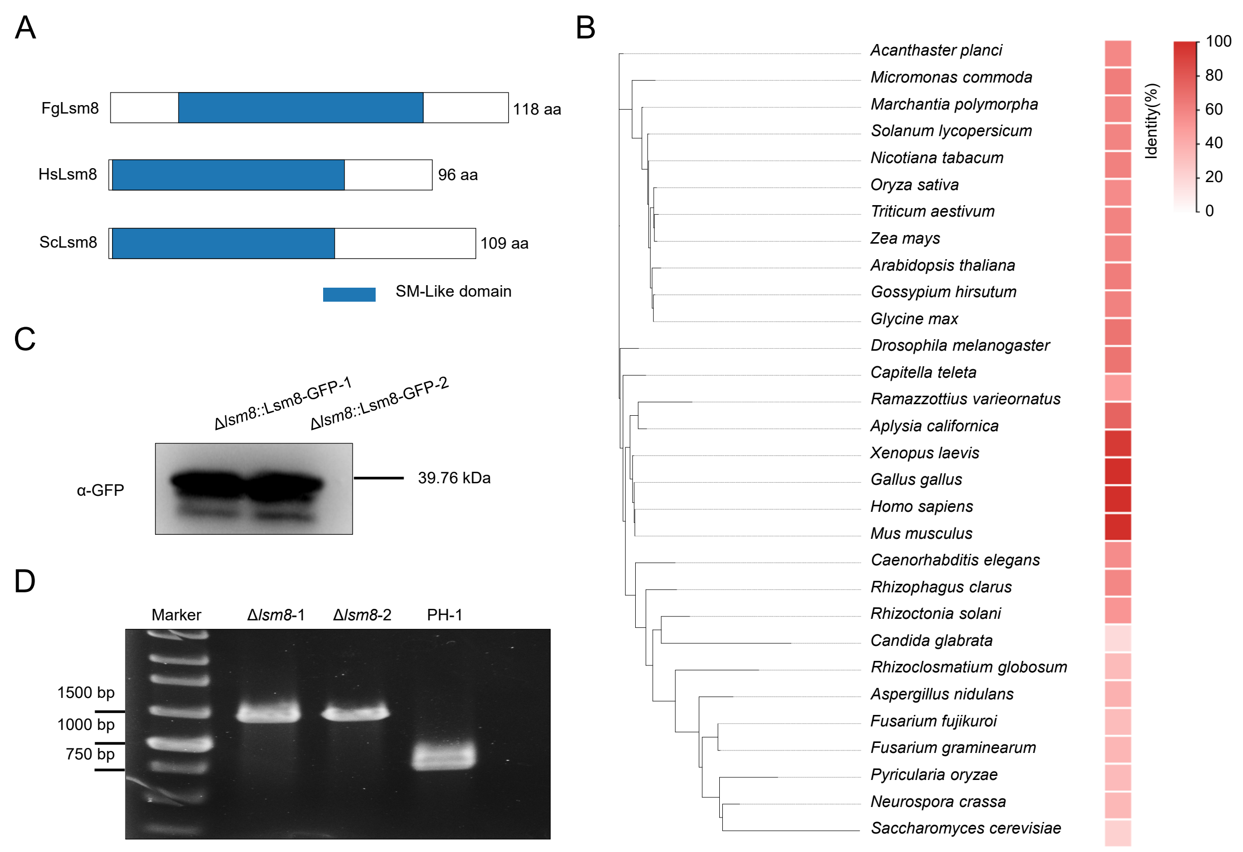

Figure S1. Molecular characterization of *F. graminearum* Lsm8 and validation of gene deletion and complementation strains. (A) Schematic representation of Like-Sm domains in Lsm8 homologs from *F. graminearum*, *Saccharomyces cerevisiae*, and *Homo sapiens*. (B) Phylogenetic tree of Lsm8 across diverse eukaryotic species, constructed using the neighbor-joining method with 1000 bootstrap replicates. *H. sapiens* Lsm8 was used as the query sequence. (C) Western blot analysis confirming the expression of the Lsm8-GFP fusion protein in the Δ*lsm8*::Lsm8-GFP complemented strain, detected with an anti-GFP antibody. The expected molecular weight is approximately 39.8 kDa. (D) PCR-based confirmation of the *LSM8* deletion in the Δ*lsm8* mutant strain.
