# Supplementary material for: A conserved Lsm8–exosome module maintains RNA splicing fidelity to control fungal stress adaptation and virulence
Source: Stress Biol. 2026 Feb 10;6(1):14. doi: 10.1007/s44154-026-00285-6 (PMC12886710; doi:10.1007/s44154-026-00285-6)
Supplement: Supplementary file 2 — Supplementary Material 2: Figure S2. Lsm8 regulates pre-mRNA splicing of ATF1 in F. graminearum. [file 44154_2026_285_MOESM2_ESM.pdf]

Figure S2

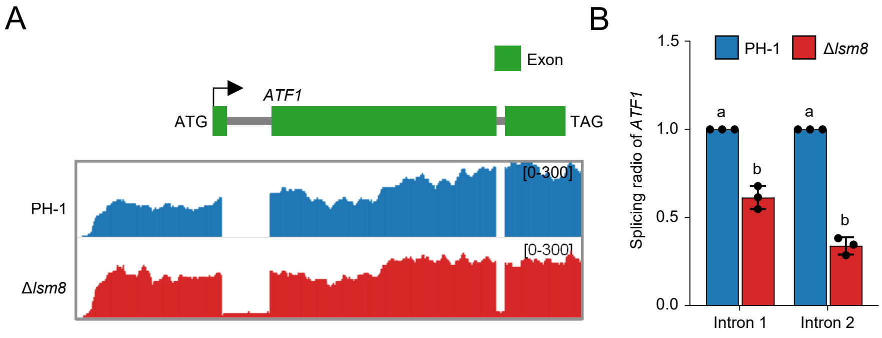

Figure S2. Lsm8 regulates pre-mRNA splicing of *ATF1* in *F. graminearum*. (A) Schematic diagram of *ATF1* gene structure. (B) Splicing efficiency analysis of *ATF1* introns in PH-1 and Δ*lsm8* by RT-qPCR. Different letters indicate a significant difference ($P < 0.05$) based on one-way ANOVA followed by unpaired Student's *t*-test.
