# Supplementary material for: A conserved Lsm8–exosome module maintains RNA splicing fidelity to control fungal stress adaptation and virulence
Source: Stress Biol. 2026 Feb 10;6(1):14. doi: 10.1007/s44154-026-00285-6 (PMC12886710; doi:10.1007/s44154-026-00285-6)
Supplement: Supplementary file 3 — Supplementary Material 3: Figure S3. Intron retention and expression profiles of upregulated splicing factor and RNA degradation genes in WT and Δlsm8. [file 44154_2026_285_MOESM3_ESM.pdf]

Figure S3

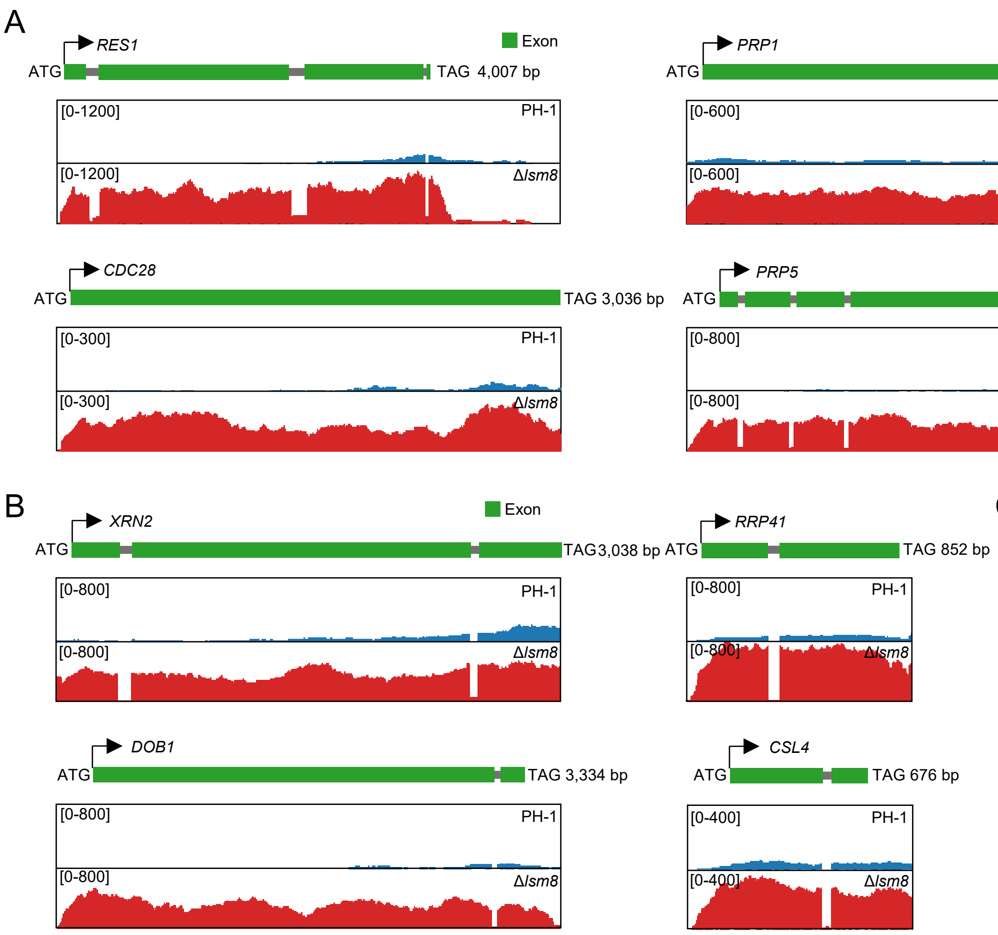

C

Gene	log_2FC	IR change
RES1	2.53	0.19,0.25,0.38
PRP1	2.11	——
CDC28	2.22	——
PRP5	2.58	0.09,0.13,0.14
XRN2	1.18	0.14,0.4
RRP4	1.6	0.05
DOB1	1.48	——
CSL4	1.00	——

Figure S3. Intron retention and expression profiles of upregulated splicing factor and RNA degradation genes in WT and Δ*lsm8*. (A) IGV visualization of RNA-seq data showing intron retention of splicing factor and (B) RNA degradation genes in Δ*lsm8* compared to PH-1. (C) log_2FC and IR change of gene in (A) and (B) in Δ*lsm8* compared to PH-1.
