# Supplementary material for: A conserved Lsm8–exosome module maintains RNA splicing fidelity to control fungal stress adaptation and virulence
Source: Stress Biol. 2026 Feb 10;6(1):14. doi: 10.1007/s44154-026-00285-6 (PMC12886710; doi:10.1007/s44154-026-00285-6)
Supplement: Supplementary file 4 — Supplementary Material 4: Figure S4. Phenotypes and subcellular localization of Xrn1 and Xrn2 in F. graminearum. [file 44154_2026_285_MOESM4_ESM.pdf]

Figure S4

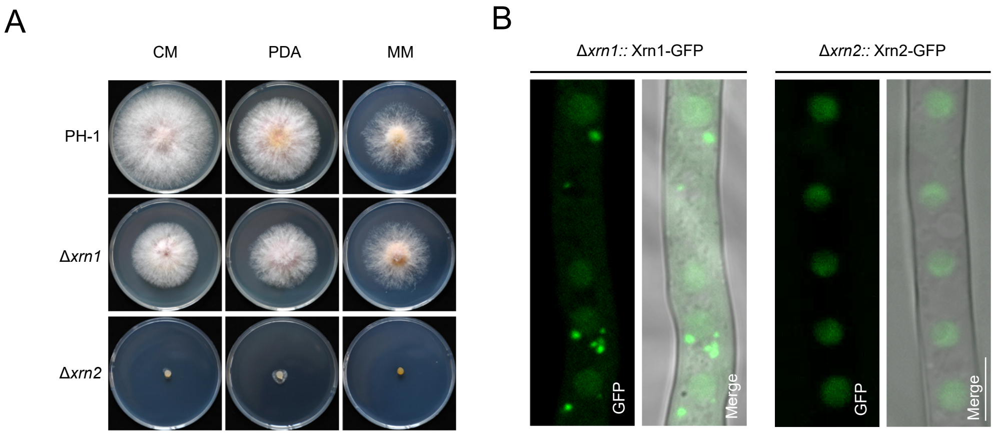

Figure S4. Phenotypes and subcellular localization of Xrn1 and Xrn2 in *F. graminearum*. (A) Colony morphology of the wild-type strain, Δ*xrn1* and Δ*xrn2* grown on PDA, MM, and CM plates for 3 days. (B) Fluorescence microscopy showing the distinct localization patterns of Xrn1-GFP and Xrn2-GFP, Scare bar=5 μm.
