# Supplementary material for: A conserved Lsm8–exosome module maintains RNA splicing fidelity to control fungal stress adaptation and virulence
Source: Stress Biol. 2026 Feb 10;6(1):14. doi: 10.1007/s44154-026-00285-6 (PMC12886710; doi:10.1007/s44154-026-00285-6)
Supplement: Supplementary file 5 — Supplementary Material 5: Figure S5. RT-qPCR primer design for distinguishing spliced and intron-retained transcripts. [file 44154_2026_285_MOESM5_ESM.pdf]

Figure S5

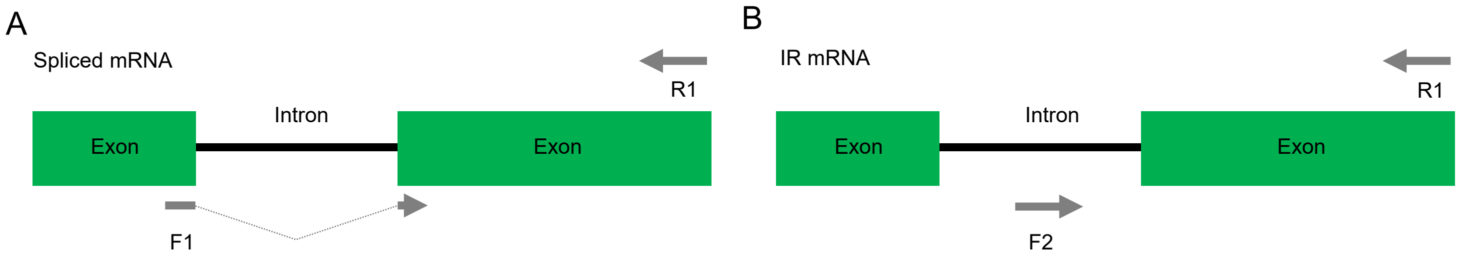

Figure S5. RT-qPCR primer design for distinguishing spliced and intron-retained transcripts. Schematic illustrating two RT-qPCR strategies used to quantify splicing outcomes. (A) For spliced transcript detection, primer F1 spans the exon–exon junction (10 bp upstream + 10 bp downstream of the intron boundary), ensuring amplification only from fully spliced mRNA, together with the downstream exon primer R1. (B) For IR transcript detection, the intron-specific forward primer F2, paired with R1, selectively amplifies unspliced mRNA species retaining the intron.
